# Bedside dressing changes for open abdomen in the intensive care unit is safe and time and staff efficient

**DOI:** 10.1186/s13054-016-1337-y

**Published:** 2016-05-28

**Authors:** Arne Seternes, Sigurd Fasting, Pål Klepstad, Skule Mo, Torbjørn Dahl, Martin Björck, Arne Wibe

**Affiliations:** Department of Vascular Surgery, St. Olavs Hospital, Trondheim University Hospital, Prinsesse Kristinas gate 3, 7030 Trondheim, Norway; Department of Anesthesiology and Intensive Care Medicine, St. Olavs Hospital, Trondheim University Hospital, Prinsesse Kristinas gate 3, 7030 Trondheim, Norway; Department of Gastrointestinal Surgery, St. Olavs Hospital, Trondheim University Hospital, Prinsesse Kristinas gate 3, 7030 Trondheim, Norway; Department of Surgical Sciences, Section of Vascular Surgery, Uppsala University, 751 85 Uppsala, Sweden; Department of Circulation and Medical Imaging, Norwegian University of Science and Technology (NTNU), Høgskoleringen 1, 7491 Trondheim, Norway; Department of Cancer Research and Molecular Medicine, Norwegian University of Science and Technology (NTNU), Høgskoleringen 1, 7491 Trondheim, Norway

**Keywords:** Abdominal compartment syndrome, Open abdomen, Dressing changes, Infections, Intensive care, Resources, Health economy

## Abstract

**Background:**

Patients with an open abdomen (OA) treated with temporary abdominal closure (TAC) need multiple surgical procedures throughout the hospital stay with repeated changes of the vacuum-assisted closure device (VAC changes). The aim of this study was to examine if using the intensive care unit (ICU) for dressing changes in OA patients was safe regarding bloodstream infections (BSI) and survival. Secondary aims were to evaluate saved time, personnel, and costs.

**Methods:**

All patients treated with OA in the ICU from October 2006 to June 2014 were included. Data were retrospectively obtained from registered procedure codes, clinical and administrative patients’ records and the OR, ICU, anesthesia and microbiology databases. Outcomes were 30-, 60- and 90-day survival, BSI, time used and saved personnel costs.

**Results:**

A total of 113 patients underwent 960 surgical procedures including 443 VAC changes as a single procedure, of which 165 (37 %) were performed in the ICU. Nine patients died before the first scheduled dressing change and six patients were closed at the first scheduled surgery after established OA, leaving 98 patients for further analysis. The mean duration for the surgical team performing a VAC change in the ICU was 63.4 (60.4–66.4) minutes and in the OR 98.2 (94.6–101.8) minutes (*p* < 0.001). The mean duration for the anesthesia team in the OR was 115.5 minutes, while this team was not used in the ICU. Personnel costs were reduced by €682 per procedure when using the ICU. Forty-two patients had all the VAC changes done in the OR (VAC-OR), 22 in the ICU (VAC-ICU) and 34 in both OR and ICU (VAC-OR/ICU). BSI was diagnosed in eight (19 %) of the VAC-OR patients, seven (32 %) of the VAC-ICU and eight (24 %) of the VAC-OR/ICU (*p* = 0.509). Thirty-five patients (83 %) survived 30 days in the VAC-OR group, 17 in the VAC-ICU group (77 %) and 28 (82 %) in the VAC-OR/ICU group (*p* = 0.844).

**Conclusions:**

VAC change for OA in the ICU saved time for the OR team and the anesthesia team compared to using the OR, and it reduced personnel costs. Importantly, the use of ICU for OA dressing change seemed to be as safe as using the OR.

## Background

Treatment of patients with open abdomen (OA) is demanding for the intensive care unit (ICU) and the hospital. OA patients require long ICU and hospital stays with repeated intra-hospital transport to the operating room (OR) for dressing changes and other surgical procedures related to the OA and/or the primary disease [1–5].

Although the well-equipped OR is the ideal location for surgery, several studies have reported that procedures like diagnostic laparoscopy, percutaneous tracheostomy, inferior vena cava filter placement, and percutaneous gastrostomy placement can be safely performed in the ICU [6–9]. Moreover, surgery done outside the OR for trauma care is also reported to be feasible [10–14]. Critical incidents occurring during intra-hospital transportation of ICU patients have been reported, and using the ICU as an OR can eliminate this problem [15, 16].

The feasibility of using the ICU as the location for planned dressing changes for OA has been demonstrated [17, 18]. The availability of OR time may be limited and planned procedures are often delayed. One potential benefit of performing dressing changes in the ICU is that it can be done during office hours, with more dedicated surgeons present, and without interfering with more urgent emergency surgery needing a fully equipped OR. Poorer outcomes of surgical and ICU treatment performed outside office hours are reported, e.g., increased mortality after treatment for ruptured aortic aneurysm [19], increased risk for anastomotic leakage of colorectal anastomosis [20], and in patients with acute traumatic coagulopathy after-hours care was associated with worse outcomes [21]. Thus, OA procedures performed in the ICU might benefit from the procedures being performed during the day shift. No previous studies have compared OA dressing changes performed in the OR versus the ICU.

According to the EPIC II study, approximately 12 % of the ICU patients die [22] and bloodstream infection (BSI) is a contributor to death [23]. Vidal et al. reported that 21 % of ICU patients with intra-abdominal hypertension had a BSI [24], and in a study of patients with OA due to abdominal compartment syndrome (ACS) following pancreatitis, 66 % had a BSI [25]. In trauma patients with ACS, BSI was reported in 26–36 % of the cases [26, 27]. The ICU is often a contaminated environment, and it may be that the risk for BSI is increased by performing the surgery in the ICU.

The primary aims of this study were to assess if using the ICU for planned OA dressing is safe for ICU patients with regard to 30-, 60- and 90-day survival and incidence of BSI compared to using the OR. Secondary aims were to evaluate if this approach saved time, personnel resources, and reduced costs.

## Methods

The study was performed in the ten-bed mixed-case ICU at St. Olavs University Hospital, Trondheim, Norway; a tertiary referral center for a population of 710,000 inhabitants. All patients treated in the ICU with OA between October 2006 and June 2014 were identified through the hospital’s patient administrative system and several departments’ specific prospective registries. Searches were also performed in the surgical procedures registry, ICU registry, anesthesia registry and in patients’ records to identify the exact surgical procedures performed on this cohort. The study was approved by the Regional Ethics Committee Mid-Norway, reference 2014/957. All living patients gave their written informed consent while the regional ethics committee waived obtaining informed consent from relatives of deceased patients.

The location of where the surgery took place (ICU, OR), type of surgical procedure, hospital length of stay, ICU length of stay, gender, age, simplified acute physiology score (SAPS II), reason for OA treatment, respirator time, and survival were obtained from patients’ records, the anesthesia registry and the ICU registry. Data on BSI were obtained from the microbiological registry. Surgical reports obtained from the patients’ records were reviewed to identify procedures involving only vacuum-assisted closure (VAC) change for OA, a procedure which was performed with a similar surgical technique in the OR and the ICU. The cohort was divided in three groups based on the location of the VAC change. The VAC-OR group having all their dressing changes done in the OR; the VAC-ICU group having all their dressing changes done in the ICU; and the VAC-OR/ICU group having dressing changes done both in the OR and ICU in no systematic order. Survival and incidence of BSI were compared between groups.

For all patients, the time used for each VAC change was obtained from the surgical and anesthesia registries. The following time-related parameters were extracted: time used by the surgical team to prepare the patient before surgery; time for the surgical procedure (“knife time”); time used by the surgical team after surgery; and total surgical team time. Anesthesia time was defined as the time used by the anesthesia team handling the patient before, during, and after surgery, including the time used to transport the patient between the ICU and the OR.

Office hours were defined as surgery taking place between 8 am and 5 pm, Monday to Friday. The time between 5 pm and 8 am and Saturdays and Sundays were defined as out of office hours.

Data on SAPS II and ICU treatment with respiratory support, dialysis, and length of stay (LOS) were obtained from the ICU registry. Date and cause of death were collected from the patients’ records. BSI was registered at the date the microbe was first identified in the blood culture. Only positive blood cultures found after initiating the OA were used in the analyses. Blood samples were not drawn as part of a scheduled plan or at a predefined time after operations, but on clinical indications.

Dressing changes (DC) for the OA included negative pressure wound therapy (NPWT) and rectus fascial traction with a mesh [4, 18, 28]. After removal of the old dressing, a new plastic film was placed between the viscera and the abdominal wall to prevent formation of adhesions to the abdominal wall and to protect the intestines from the foam. An outer sponge secured by a plastic drape covered the abdominal defect. Vacuum was applied at a continuous negative pressure of 50 to 125 mm Hg, both V.A.C.® therapy and ABThera™ (KCI, San Antonio, TX, USA) were used. According to the standardized protocol, the dressing was removed and the abdominal wall closed provided this could be done without tension after 2 or 3 days. If closing was not possible, a new dressing change was performed. The OA protocol requires change of dressing every second or third day, earlier if necessary due to alteration of the patient’s condition. No protocol existed for where the dressing changes should take place, and the decision of using either the OR or ICU was done by the surgical team in care of the patient based on their preferences.

**Fig. 1:**
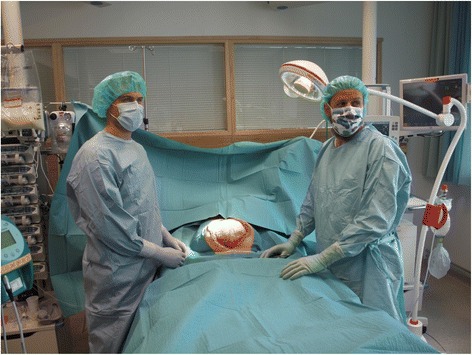
Performing the dressing change in the intensive care unit (ICU) in a sterile fashion with a portable operating light

When surgery was performed in the OR, a fully equipped OR was used involving two surgeons, one surgical nurse, one scrub nurse, one anesthetist and two nurse anesthetists, engaging a total number of seven health workers. After use, the OR was cleaned and prepared in order for the next procedure by two cleaners taking 30 minutes each. The changes were done with either general or regional anesthesia. VAC change at ICU was performed with a team of two surgeons, one surgical nurse and one scrub nurse in addition to the ICU nurse. The already intubated patient was given opioids, sedatives and muscle relaxants as ordered by the ICU physician, administered by the ICU nurse. All personnel in the room used a surgical cap and mask; those in the field scrubbed in and used sterile operating garments and gloves (Fig. 1). Only a small surgical kit with the necessary equipment for completing the VAC change was used. Admittance to the operating field was restricted, and the door, if any, was closed and guarded. No equipment for bowel resection/major surgery was present, but if necessary, it could be available in a few minutes, or if in need for more extensive surgery, a temporary abdominal closure (TAC) was performed and the patient transferred to an ordinary OR for completion of the surgery.

All emergency surgery is prioritized to OR according to a traffic light coding system, modified from Leppäniemi et al. [29]. Patients were classified as red, yellow, and green, which correspond to a maximum of 6, 24, and 72 hours delay before surgery. Initial treatment for ACS is defined as red and VAC change for the OA is defined as yellow.

The personnel costs were estimated from average wages with social benefits for the year of 2014 for each profession involved. The costs for 1 hour with an anesthetist and a surgeon is €98 each, for a scrub nurse and a nurse anesthetist €65 each and for the cleaners €40 each. The mean elapsed time for each of the personnel groups involved in the procedure was used for the calculation.

### Statistics

Continuous data are presented as median with range or mean with 95 % confidence interval (CI). Between-group comparisons of continuous variables were performed with Mann-Whitney test (nonparametric) or Student’s *t* test and one-way analysis of variance (ANOVA) (parametric), and if extreme skewness transformation was used. Statistical comparisons of the duration of the VAC change, including total time, surgical time, and duration of anesthesia in the ICU compared to the OR were performed with an independent *t* test. Categorical variables were compared using Pearson chi-square test or Fisher’s exact test. Cox regression analysis was used to perform adjusted survival analysis. The statistical significance level was set to *p* < 0.05, two-tailed. Data were analyzed in Excel, Windows 2010 (Microsoft Corp., Redmond, WA, USA) and IBM SPSS software, version 21 (IBM Corp., Armonk, NY, USA).

## Results

All 113 patients treated with OA from the Departments of Surgery (n = 95), Trauma (n = 9), Internal Medicine (n = 5), and Gynaecology and Obstetrics (n = 4) were included. Indications for OA were abdominal compartment syndrome (ACS) (n = 53), abdomen could not be closed due to intra-abdominal swelling (loss of domain) (n = 27), abdominal contamination/second look (n = 19), necrotizing fasciitis (n = 7), hemorrhage packing (n = 4), and full thickness dehiscence (n = 3). A total of 960 surgical procedures were performed, of which 443 were dressing changes and 109 were dressing changes combined with other procedures like mesh placement to complete the TAC (n = 34), and resection of ischemic bowel and gall bladder (n = 19). After the index operation for OA, nine patients died before the first scheduled DC and six patients were closed at the first scheduled surgery after OA was established, leaving 98 patients for further analysis.

These 98 patients were in need of 552 VAC changes after the index operation for open abdomen, with a median of four (range 1–26) procedures. The number of VAC changes being the only procedure was 443 with 278 done in the OR and 165 done in the ICU, among which 413 were scheduled and 30 were unplanned. Of the unplanned, 24 were done in the OR and six in the ICU. All changes at the ICU were completed as planned, except in one patient who was transferred to the OR due to an unexpected finding of necrotizing pancreatitis which needed necrosis removal. Forty-two patients had all VAC changes done at the OR (VAC-OR), 22 all in the ICU (VAC-ICU), and 34 patients had VAC change done both in the OR and ICU (VAC-OR/ICU). There were no differences in age, SAPS II, sex and ACS as reason for OA between the groups, but renal replacement therapy (RRT) was more frequent in the VAC-ICU and VAC-ICU/OR group, 32 % and 35 %, respectively, compared to 12 % in the VAC-OR group (*p* = 0.0206) (Table 1). All patients received mechanical ventilator support, and most of them until their abdomens were closed. Nineteen of the patients were re-intubated for a median of three (1–19) procedures before closure of the open abdomen. Patients in the VAC-OR group had fewer days on respirator compared to the VAC-ICU group, 11.8 vs. 20.4 days (*p* = 0.007), respectively (Table 3). Similarly, the ICU LOS was 15 days for the VAC-OR group compared to 21.5 days in the VAC-ICU group (*p* = 0.787). However, LOS in the hospital was 35.5 days in the VAC-OR group and 34.5 days in the VAC-ICU group.

**Table 1:** Patients characteristics

	All n = 98	VAC-OR n = 42	VAC-ICU n = 22	VAC-OR/ICU n = 34	*p*
Number of men (%)	73 (72 %)	27 (64 %)	17 (77 %)	28 (82 %)	0.100^a^
Age, median (range)	64 (20–88)	58.5 (22–88)	70.5 (24–82)	65.5 (20–82)	0.093^b^
Reason for OA, n (%)					
ACS	46 (47 %)	18 (43 %)	10 (46 %)	18 (53 %)	0.681^a^
Intraabdominal swelling	25 (26 %)	9 (21 %)	9 (41 %)	7 (21 %)	0.198^a^
Abdominal contamination/second look	14 (14 %)	7 (17 %)	3 (14 %)	4 (12 %)	0.931^a^
Other	13 (13 %)	8 (19 %)	0 (0 %)	5 (15 %)	0.077^a^
Primary diagnosis, n (%)					
Vascular	45 (46 %)	14 (33 %)	14 (64 %)	17 (53 %)	0.058^a^
Gastrointestinal	31 (43 %)	18 (43 %)	4 (18 %)	9 (27 %)	0.094^a^
Trauma	9 (8 %)	3 (7 %)	1 (5 %)	5 (15 %)	0.481^a^
Urological	6 (6 %)	4 (10 %)	0	2 (6 %)	0.421^a^
Internal medicine	4 (4 %)	0	3 (14 %)	2 (3 %)	0.038^a^
Gynecological	3 (3 %)	3 (7 %)	0	0	0.240^a^
Clinical characteristics					
SAPS II, median (range)	43.1 (40.3–45.8)	40.2 (36.0–44.3)	46.6 (41.0–52.6)	44.3 (39.5–49.0)	0.158^b^
Dialysis, n (%)	24 (24 %)	5 (12 %)	7 (32 %)	12 (35 %)	0.0206^a^

*VAC-OR* all dressing changes in the operating room, *VAC-ICU* all dressing changes in the intensive care unit, *VAC-OR/ICU* dressing changes in the operating room and intensive care unit, *OR* operating room, *ICU* intensive care unit, *OA* open abdomen, *ACS* abdominal compartment syndrome, *SAPS II* simplified acute physiology score ll

^a^Fisher exact test; ^b^one-way ANOVA

The mean total time the surgical team spent on VAC change was 63.4 (60.4–66.4) minutes when using the ICU compared to 98.2 (94.6–101.8) minutes in the OR, with a difference of 33.8 (27.0–40.6) (*p* < 0.001). Time used for the anesthesia team in the OR was 115.5 (111.0–120.0) minutes (Table 2). The anesthesia team was not involved in VAC changes done in the ICU, and therefore, the time saved for the anesthesia team for three persons equals the total time used in the OR (115.5 × 3 = 346.5 minutes).

**Table 2:** Time used for open abdomen dressing changes in the intensive care unit (ICU) and operating room (OR)

	ICU n = 165	OR n = 278	Time difference	*p*
Preoperative time (min)	26.4 (24.4–28.3)	45.1 (43.1–47.0)	18.7 (15.7–21.7)	<0.0001^a^
Surgical time (min)	29.8 (27.9–31.7)	35.2 (33.2–37.2)	5.5 (2.5–8.5)	<0.0001^a^
Postoperative time (min)	7.2 (6.6–7.8)	17.9 (16.5–19.3)	10.7 (9.2–12.2)	<0.0001^a^
OR/sum time (min)	63.4 (60.4–66.4)	98.2 (94.6–101.8)	33.8 (27.0–40.6)	<0.0001^a^
Anesthesia time (min)	NA	115.5 (111.0–120.0)	NA	NA

Values in minutes with mean and 95 % confidence interval.

*NA* not applicable

^a^Student’s *t* test

For a patient having the dressing change performed in the OR, the personnel costs for all employees were €908, compared to €226 when the ICU was used, thus personnel costs were reduced by €682 for each dressing change.

For VAC changes in the ICU, 122 (74 %) were performed during weekdays, similar to the 210 (76 %) procedures performed during the weekdays in the OR (*p* = 0.734). The dressing changes were performed during office hours in 93 out of 165 (56 %) ICU procedures, similar to 157 out of 278 (56 %) OR procedures (*p* = 1).

BSI was detected in 33 (29 %) patients during the hospital stay; in ten patients prior to OA treatment and in 23 patients during or after OA treatment (Table 3) with a median of 13 (range 1–96) days after established OA. No multidrug-resistant strains were found. The median time from OA being established to intestinal species being detected in the blood was 15 (2–96) days, for staphylococcal infection it was 19 (4–81) days, and for candida 8 (3–13) days. In the 70 patients surviving 90 days, 20 patients had a BSI, compared to 13 in the 28 patients not surviving 90 days (*p* = 0.103). In the 42 patients having their VAC changes done in the OR, eight patients (19 %) were diagnosed with a BSI during or after OA treatment compared with seven of 22 (32 %) patients in the VAC-ICU group, and eight of 34 (24 %) in the VAC-OR/ICU group (*p* = 0.509).

**Table 3:** Duration of open abdomen (OA), respirator and intensive care unit (ICU) treatment, number and type of bloodstream infection (BSI) and survival

	All (n = 98)	VAC-OR (n = 42)	VAC-ICU (n = 22)	VAC-OR/ICU (n = 34)	*P*
Days with OA (median, range)	13 (1–143)	10.5 (1–88)	12.5 (2–22)	18.5 (2–143)	0.002^b^
Days on respirator (median, range)	15.5 (1–62)	11.8 (1–62)	20.4 (9–49)	16.1 (1–48)	0.007^b^
LOS ICU, days (median, range)	18 (1–89)	15 (1–70)	21.5 (7–67)	17 (8–89)	0.078^b^
LOS total hospital, days (median, range)	35.5 (3–246)	35.5 (3–215)	34.5 (10–143)	36 (10–246)	0.787^b^
Bloodstream infection (n, %)	23 (23 %)	8 (19 %)	7 (32 %)	8 (24 %)	0.509^a^
*Escheria coli*		3	1	0	
*Enterococci*		0	1	4	
*Enterobacter*		1	0	0	
*Staphylococci*		3	3	2	
*Candida*		0	2	1	
*Bacteroides*		1	0	0	
*Beta-hemolytic streptococci g. A*		0	0	1	
30-day survival (n, %)	80 (82 %)	35 (83 %)	17 (77 %)	28 (82 %)	0.844^a^
60-day survival (n, %)	73 (75 %)	34 (81 %)	16 (73 %)	23 (68 %)	0.384^a^
90-day survival (n, %)	70 (71 %)	34 (81 %)	15 (68 %)	21 (62 %)	0.172^a^

*VAC-OR* all dressing changes in the operating room, *VAC-ICU* all dressing changes in the intensive care unit, *VAC-OR/ICU* dressing changes in the operating room and intensive care unit, *OR* operating room, *ICU* intensive care unit, *OA* open abdomen, *LOS* length of stay

^a^Fisher exact test; ^b^one-way ANOVA

Eighty patients (82 %) survived 30 days. Thirty-five patients (83 %) survived in the VAC-OR group, 17 (77 %) in the VAC-ICU group and 28 (82 %) in the VAC-OR/ICU group (*p* = 0.844). The 60- and 90-day survival rates were 75 % and 71 % respectively, with no difference between the subgroups (Table 3).

**Fig. 2:**
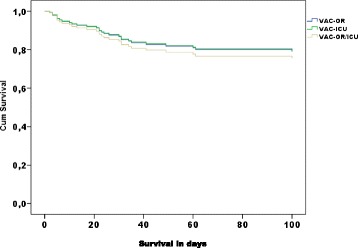
Cox regression analyses for survival dependent on where dressing change where performed adjusted for age, sex, renal replacement therapy, simplified acute physiology score II (SAPS II) and incidence of bloodstream infection (BSI). *VAC-ICU* all dressing changes in the intensive care unit, *VAC-OR* all dressing changes in the operating room, *VAC-OR/ICU* dressing changes in the operating room and intensive care unit

In a multivariate analysis adjusting for age, sex, SAPS II, dialysis and location of dressing change, only high age and need of renal replacement therapy increased the hazard ratio (HR) of death with 1.04 (95 % CI: 1.001–1.081 %, *p* = 0.047) and 2.47 (95 % CI 1.08–5.65, *p* = 0.032), respectively (Fig. 2).

## Discussion

This study demonstrated that VAC change on patients with open abdomen (OA) can be done safely outside the operating room. Utilizing the ICU as a surgical suite for performing repeated changes of the OA did neither influence 30-, 60- and 90-day survival nor incidence of BSI. Additionally, the study showed that performing the dressing change in the ICU reduced costs and time spent on the surgical procedure, and it made the anesthesia team superfluous.

High age and renal replacement therapy were associated with an adverse outcome after open abdomen treatment [30]. Other studies have reported survival after OA therapy in the range of 50–72 %. Thus, the survival of patients with OA in the present study was similar to previous reports [2, 4, 5, 17, 31–33]. The VAC-ICU patients stayed longer at the ICU compared to the VAC-OR group, most likely due to more severe pulmonary and renal failure, however, the length of stay in the hospital and survival were similar.

Despite the risk of a more contaminated ICU environment for the patients with DC performed in the ICU, they were not at a higher risk of BSI during and after the OA treatment. BSI affected almost one third of the study population, and in the patients not surviving 90 days almost half had BSI. This observation is in line with previous studies including patients with abdominal hypertension, ACS or OA [24, 26, 27], and eight of the 23 BSIs were due to staphylococcal infection, and thus most likely they were caused by intravenous catheters and not by contamination from the OA.

To our knowledge, this is the first study comparing time spent for VAC change on OA for two surgical locations; the OR and the ICU. The present results demonstrate a significant reduction of the time spent on preparing the OA patient for surgery when the VAC changes were performed at the ICU. The excess time used before surgery at the OR did not only relate to transportation from the ICU to the OR, but also to the time used to move the patients from the bed to the OR table, and also the time used when more personnel groups are involved. One example of the latter is the use of special assistants to lift and position the patients at the OR table at our hospital. The difference in time used for the procedure could be addressed by for instance preparing a simplified surgical equipment package similar to the one used in the ICU instead of the more advanced surgical equipment package used in the OR. The surprising finding that surgical time (knife time) differed in favor of having the dressing changed in the ICU was not due to more advanced surgery performed at the OR, as similar procedures were compared. Furthermore, the same surgeons and nurses were involved in the procedures at both locations. The time difference may partly be explained by the fact that all surgical equipment was immediately available in a prepared surgical kit in the ICU room. The time used after the procedure was finished was also significantly shorter in the ICU group, due to no need of patient transportation and less use of surgical equipment. Importantly, the anesthesia team was not involved in the treatment performed in the ICU, making an entire anesthesia team available for other activities. Altogether, the use of the ICU saved considerable personnel costs for the hospital.

Only one patient who had his dressing changed at the ICU needed to be transferred to the OR for completion of surgery. In all other cases, the dressing change was completed in the ICU. This supports a practice where OA dressing changes can be done in the ICU as long as no additional procedures are planned.

The organization of emergency surgery is important, as the availability of surgical teams, anesthesia teams and ORs are limited resources. VAC changes for OA can either delay emergency surgeries or necessitate VAC change for OA to be done after office hours. Moreover, this group of patients is usually complex, needing ventilator support and multiple infusions including vasoactive drugs. The unstable patients is exposed to a substantial risk when being transferred out of the ICU to the OR, which should be avoided if not clearly indicated [15, 16, 34]. Of course patients need to be monitored during the DC, and most patients need additional analgesics, sedatives and muscle relaxants during the procedure, but this can be administered by ICU personnel caring for the patient in the ICU.

We recognize that this study has limitations. This was a retrospective study and there was no predefined protocol to decide where to perform the DCs. Therefore, a bias may have been introduced as the surgical team performing the DC chose the location based on their preference and/or the patient’s condition, introducing multiple possible confounding factors. For instance, more patients in the ICU and ICU/OR groups received dialysis compared with the OR group. This may reflect that dressing changes in those patients were done in the ICU in order not to interrupt continuous renal placement therapy. Furthermore, the blood cultures were obtained as indicated and not routinely collected, and other infections such as local infections in the OA were not included in the data material. Although the current study was relatively large compared to other publications, the numbers are still limited for each subgroup, and therefore, due to the risk of type II statistical errors, the results should be interpreted with caution. Finally, this is a single-center study, and all findings may not be generalizable to other organizations. Hence, larger cohorts, preferably multicenter studies, with standardized assessments of complications are needed in order to conclude on outcomes related to location for dressing change for OA treatment.

## Conclusions

In this study on 98 patients, VAC changes for open abdomen in the ICU were cost and time efficient for the surgical and anesthesia departments, and seemed to be safe. Further studies on larger patient cohorts, preferably with a prospective multicenter design, are warranted.

## Key messages

- Performing VAC changes for open abdomen in the ICU is cost, time, and staff efficient.

## Abbreviations

ACS, abdominal compartment syndrome; ANOVA, analysis of variance; BSI, bloodstream infection; CI, confidence interval; DC, dressing change; HR, hazard ratio; ICU, intensive care unit; LOS, length of stay; NPWT, negative pressure wound therapy; OA, open abdomen; OR, operating room; RRT, renal replacement therapy; SAPS II, simplified acute physiology score II; TAC, temporary abdominal closure; VAC, vacuum-assisted closure
